# Predicting HIV infection in the decade (2005–2015) pre-COVID-19 in Zimbabwe: A supervised classification-based machine learning approach

**DOI:** 10.1371/journal.pdig.0000260

**Published:** 2023-06-07

**Authors:** Rutendo Beauty Birri Makota, Eustasius Musenge

**Affiliations:** Division of Epidemiology and Biostatistics, School of Public Health, Faculty of Health Sciences, University of the Witwatersrand, Johannesburg, South Africa; Harvard University T H Chan School of Public Health, UNITED STATES

## Abstract

The burden of HIV and related diseases have been areas of great concern pre and post the emergence of COVID-19 in Zimbabwe. Machine learning models have been used to predict the risk of diseases, including HIV accurately. Therefore, this paper aimed to determine common risk factors of HIV positivity in Zimbabwe between the decade 2005 to 2015. The data were from three two staged population five-yearly surveys conducted between 2005 and 2015. The outcome variable was HIV status. The prediction model was fit by adopting 80% of the data for learning/training and 20% for testing/prediction. Resampling was done using the stratified 5-fold cross-validation procedure repeatedly. Feature selection was done using Lasso regression, and the best combination of selected features was determined using Sequential Forward Floating Selection. We compared six algorithms in both sexes based on the F1 score, which is the harmonic mean of precision and recall. The overall HIV prevalence for the combined dataset was 22.5% and 15.3% for females and males, respectively. The best-performing algorithm to identify individuals with a higher likelihood of HIV infection was XGBoost, with a high F1 score of 91.4% for males and 90.1% for females based on the combined surveys. The results from the prediction model identified six common features associated with HIV, with total number of lifetime sexual partners and cohabitation duration being the most influential variables for females and males, respectively. In addition to other risk reduction techniques, machine learning may aid in identifying those who might require Pre-exposure prophylaxis, particularly women who experience intimate partner violence. Furthermore, compared to traditional statistical approaches, machine learning uncovered patterns in predicting HIV infection with comparatively reduced uncertainty and, therefore, crucial for effective decision-making.

## Author summary

The Joint United Nations Programme (UNAIDS) set up fast-track targets to reach HIV epidemic control by 2030, where it is expected that 95% of people living with HIV know their status, and of those, 95% should be on treatment and of those on treatment 95% should have reached viral suppression. In Zimbabwe, by 2020, it was found that 86.8% of adults living with HIV were aware of their status and of those aware of their status, 97.0% were on antiretroviral treatment. Furthermore, among those on treatment, 90.3% achieved viral load suppression. In order to achieve these targets, modern predictive algorithms using machine learning have the power to enhance HIV prevention and prediction capability. Furthermore, studies have reported that machine learning could accurately predict future HIV infection. This paper, therefore, aims to use these machine learning tools to predict key HIV populations based on socio-behavioural characteristics obtained from survey data.

## Introduction

In the era before and after COVID-19, HIV ranks amongst the most serious infectious diseases globally. Despite the tremendous advancements in diagnosis and access to antiretroviral therapy (ART), Zimbabwe Population-based HIV Impact Assessment survey (ZIMPHIA 2020) predictions suggest that 1.23 million adults are living with HIV and that the incidence rate is about 0.21% annually [1]. HIV/AIDS as a threat to the public’s health was to be eradicated by 2030, according to the Joint United Nations Programme (UNAIDS) [2,3]. The COVID-19 pandemic, however, is already reversing the gains obtained, and it may have a negative impact by increasing the number of AIDS-related fatalities in sub-Saharan Africa [4,5]. Furthermore, the HIV epidemic is unevenly spread throughout several geographic locations, with higher prevalence in some areas and among particular populations. Focused interventions are necessary for this situation to manage the HIV pandemic effectively since they have been found to maximise the prophylactic benefit at the lowest possible cost [6]. Therefore, it is essential to understand where and among which groups new infections develop to inform targeted interventions.

Monitoring and surveillance techniques, such as behavioural risk assessments, interviews, and laboratory test results, should form the basis of a nation’s HIV response [7]. Unfortunately, most nations’ surveillance systems, including structured government surveillance and ad hoc surveys, are insufficient to accurately monitor epidemic and risk patterns [7]. In addition, there is frequently a reporting lag before data are made public, and data collecting and aggregation techniques are time- and resource-intensive [8].

Non-requirement of statistical inferences or assumptions is one advantage of machine learning algorithms for developing predictive models. Since machine learning algorithms are data-driven, their greatest benefit is their ability to automatically learn from data that identifies complex nonlinear patterns and exploits complex interactions between risk factors. Machine learning models have been used to predict the future risk of other conditions [9–14], including HIV. Studies have reported that machine learning could accurately predict future HIV infection [2,15–18].

Three primary studies have been published that used machine learning methods to predict HIV status in Zimbabwe [2,19,20]. First, Mutai et al. [2] predicted HIV status using Demographic Health Survey data from Sub-Saharan Africa. The results obtained from the study by Mutai et al. [2] were not specific to Zimbabwe but sub-Saharan Africa. Second, using data from the Zimbabwe Ministry of Health and Child Care, Chingombe et al. [19] predicted HIV status among men who had sex with men in Zimbabwe’s two major cities, Bulawayo and Harare. The findings of this study were limited to men who had sex with men in Bulawayo and Harare and could not be generalised to other cities in Zimbabwe or the general population. Third, using nationally representative data from a cross-sectional survey from the Zimbabwe Population-Based HIV Impact Assessment (ZIMPHIA15-16), again, Chingombe et. [20] predicted the HIV status using machine learning techniques. Based on the three primary studies that employed machine learning techniques to estimate the HIV status in Zimbabwe, our study and findings will add to the existing body of knowledge. In contrast to Mutai et al.’s [2] research, the findings of our study will be more applicable to Zimbabwe. In addition, unlike the two studies by Chingombe et al. [19,20], we have access to a vast quantity of data from 2005–2015, i.e., ten years of DHS data. Therefore, the primary aim of this paper was to determine common risk factors of HIV positivity in Zimbabwe. Secondarily with the aid of machine learning algorithms, these risk factors were used to formulate a model that predicts HIV positivity.

## Methodology

### Data and study design

Situated in the southern parts of Africa, Zimbabwe is a landlocked nation with a population of 5.1 million people based on the 2022 census [21]. Three nationally representative surveys were held in 2005–06, 2010–11, and 2015 and all named Zimbabwe Demographic Health Survey (ZDHS). The data utilised for analysis in this paper can be obtained from the Demographic Health Survey (DHS) programme website (https://dhsprogram.com) [22]. Since 1984, around 70 nations have conducted DHS, which are nationally representative household surveys [23–25]. In the fields of population, health, and nutrition, they offer data for various monitoring and impact evaluation. Accordingly, blood samples were taken in all households with the consent of the respondent or parent/guardian (for minors) for HIV testing in the lab for females aged 0–49 and males aged 0–54. This was a retrospective cross-sectional study design that employed secondary data analysis.

### Data pre-processing

**Fig 1:**
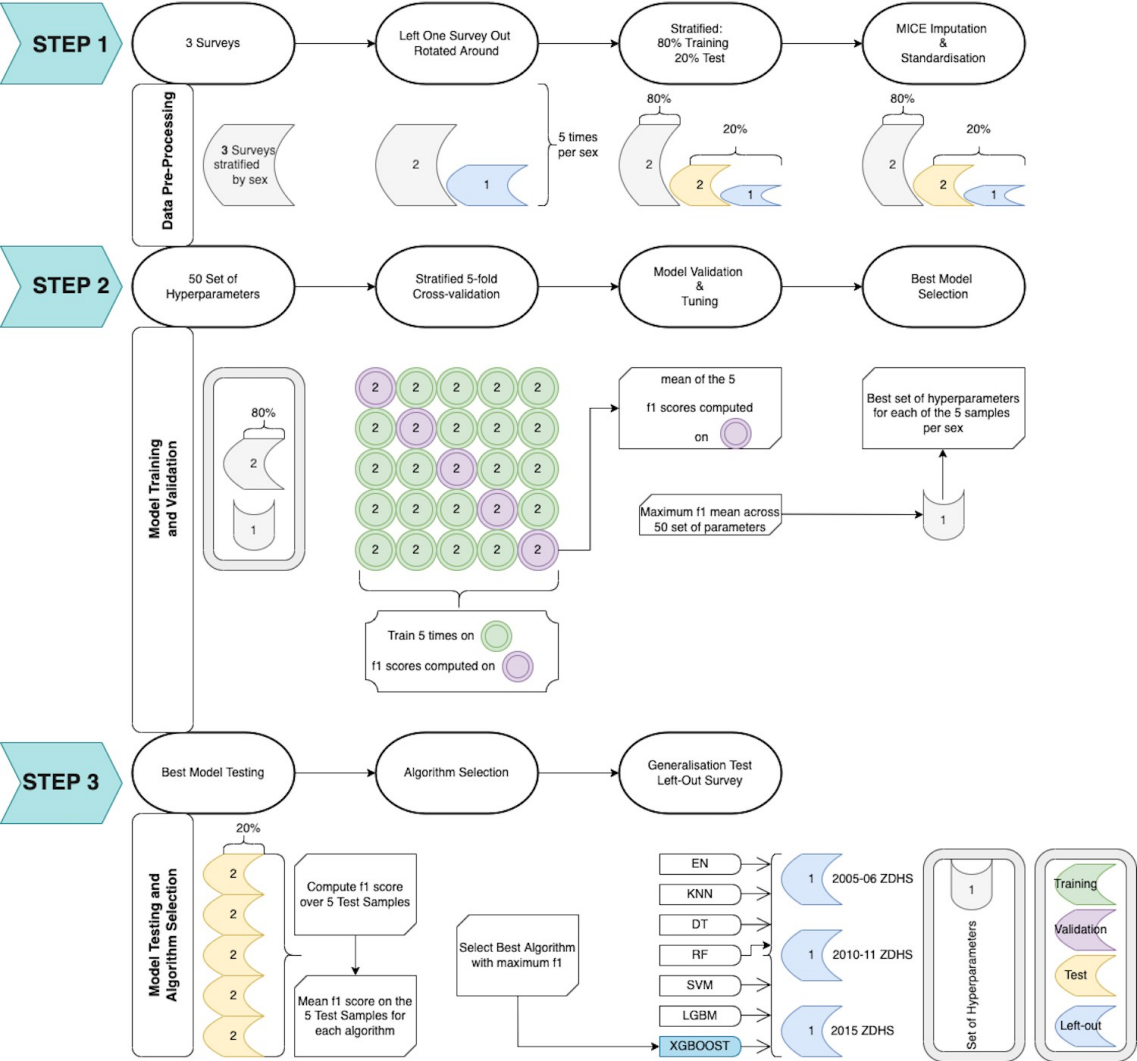
Flowchart of the methods and steps used.

The datasets from the three ZDHS HIV test results and the datasets from adult interviews were merged, and records without an HIV test result were excluded from the analysis. The outcome variable, HIV status, was divided into two categories (0 for HIV negative and 1 for HIV positive). To account for non-response, non-coverage, and population total adjustment weights, data were resampled utilising sample weights of HIV test results for each survey year. Variables with a correlation coefficient of 80% or higher, non-unique columns, arbitrary features, more than 30% missing values, and uninformative features were all eliminated. The label-code and one-hot encoding methods were used to encode nominal and ordinal variables derived from survey data. As part of step 1, represented in Fig 1, missing values were imputed using Multiple Imputations with Chained Equations (MICE), and in each of these categories, we assumed that missing was at random. The data were further normalised and scaled.

### Train, test and validation procedure

The data were stratified by sex, and subsequent data training, testing and validation were done following the stratification. Following step 1 from Fig 1, one survey year was left out, with all the survey years being rotated and left out to produce three distinct datasets for each sex, each with only two surveys. The primary goal of the rotation was to evaluate the generalisation of the models separately for males and females in later testing and validation. Of the three newly constructed datasets for each sex as shown in Fig 1 step 1, 80% were selected for training, and 20% were utilised as test and validation samples. To prevent the test dataset from being contaminated, MICE imputation and data standardisation were carried out independently for the training and test datasets.

### Feature selection

Feature selection was done in two stages. We first used LASSO (Least Absolute Shrinkage and Selection Operator) regression to select features important in determining HIV status using combined dataset from the three surveys. Features selected through LASSO regression where then used to fit the algorithms under investigation. On variables selected through LASSO regression, subsequent analysis utilised the method with the algorithm with the highest F1 score and analysis was done separately for each survey year stratified by sex. We utilised the sequential forward floating selection (SFFS) approach with 80% of the training samples to select the final features to use in best algorithm among the six algorithms. Feature selection using the SFFS was implemented by considering features whose F1 score plateaued from the saturation point. Using SHapley Additive exPlanations (SHAP) [26], the contribution of each feature selected through SFFS to the probability of being HIV-positive was then examined.

### Data balancing

Considering that the proportion of those HIV negative and positive was imbalance with a ratio of 4.2:1, we applied resampling methods to handle the class imbalance. We performed the Synthetic Minority Over-sampling Technique (SMOTE) to balance the classes. This was achieved by generating synthetic data using the nearest neighbour’s algorithm to balance out the classes. Original dataset without implementing the SMOTE procedure was also analysed to compare results.

### Machine learning models

A supervised machine learning binary classification was implemented. The following machine learning algorithms were compared: a penalised logistic regression (Elastic Net), k-nearest neighbour algorithm (KNN), Random Forest Classifier (RFC), Decision Tree approach (DT), Light Gradient Boosting Model (LightGBM) and the XGBoost model. To achieve step 2, training datasets were used, and hyperparameter tuning from a grid of 50 sets was performed using a randomised grid search. Then, using the stratified five-fold cross-validation technique over the validated samples, the average F1 scores for each of these 50 sets was calculated, and the most powerful set of hyperparameters was chosen.

Each of the best three models (for each survey year) by sex and algorithm was then run on the corresponding test dataset described in Step 3, Fig 1, and the resulting metric scores were averaged. Next, the algorithm with the best average F1 score was selected from the three test datasets. Finally, each selected model was applied to the survey left-out dataset.

### Algorithm evaluation

Due to the unequal class distribution of the classification variable with a ratio of 4.2:1 in the original dataset, the Precision-Recall curve is recommended over the Receiver Operating Characteristic (ROC) curve [27] due to its insensitivity to imbalanced datasets. Other metrics were also considered to evaluate the algorithm’s performance: accuracy, F1, precision and recall scores. To determine the ratio of correct classification, accuracy score was used. To determine the prediction of true positive cases, precision was used. To determine the proportion of positive cases that are correctly predicted, recall score was used. Lastly, the F1 score which is the weighted harmonic mean of recall and precision was used to determine the predictive power of the algorithm. An algorithm with the highest F1 score was considered to have the best predictive power. Finally, each evaluation used the same segmentation and repetition of data to ensure a fair comparison of models.

### Statistical analysis

The features that were chosen using the SFFS XGBoost procedure were subjected to a logistic regression analysis. This step was added to confirm the outcomes that the SHAP plots yielded. In addition to the results of the SHAP plots, the logistic regression also provided the magnitude and direction of the risk of contracting HIV.

## Results

### Summary statistics

The overall HIV prevalence for the combined dataset, as shown in Table 1, was 22.5% and 15.3% for females and males, respectively. Those who were not currently employed had a higher prevalence (54.1%) than those who were currently employed (45.9%) for females. However, the opposite relationship is seen in males, where those currently employed had a higher HIV prevalence (74.2%) than those not currently employed (25.8%). Adults had a higher HIV prevalence than youths for both sexes. Of interest was a higher HIV prevalence noticed in those who had the highest level of education as secondary for both sexes.

**Table 1:** Background characteristics of ZDHS data.

	*FEMALES*	*MALES*
***Characteristic***	**HIV Positive n(%)**	**HIV Positive n(%)**
*Overall summary*	4457(22.5)	2215(15.3)
***Survey year*:** *2005–06*	1604(36.0)	760(34.3)
*2010–11*	1332(29.9)	733(33.1)
*2015*	1521(34.1)	722(32.6)
***Current age*:** *youths (15–24 years)*	730(16.4)	179(8.1)
*Adults (25–54 years)*	3727(83.6)	2036(91.9)
***Marital Status***: *single*	302(6.8)	183(8.3)
*cohabiting/married*	2595(58.2)	1723(77.8)
*Divorced/separated/widowed*	1560(35.0)	309(14.0)
***Place of Residence*:** *urban*	1625(36.5)	877(39.6)
*rural*	2832(63.5)	1338(60.4)
***Education level*:** *no education*	123(2.8)	23(1.0)
*primary*	1510(33.9)	612(27.6)
*secondary*	2674(60.0)	1431(64.6)
*higher*	150(3.4)	149(6.7)
***STI treatment in past 12 months***: *no*	4127(92.7)	2102(94.9)
*yes*	325(7.3)	113(5.1)
***Number of unions***: *once*	2942(70.9)	1059(52.2)
*more than once*	1210(29.1)	970(47.8)
***Currently employed***: *no*	2410(54.1)	571(25.8)
*yes*	2047(45.9)	1644(74.2)
***Wealth index***: *poorest*	781(17.5)	366(16.5)
*poorer*	727(16.3)	411(18.6)
*middle*	929(20.8)	350(15.8)
*richer*	1198(26.9)	603(27.2)
*richest*	822(18.4)	485(21.9)

### Feature selection

LASSO regression was performed to determine the possible predictors of HIV infection. A total of 32 predictors were considered for both males and females (see S1 Table). Out of the 32 predictors, type of place of residence, religion, number of household members, relationship to household head, sex of household head, age of household head, times away from home in last 12 months, wealth index, current contraceptive method, current contraceptive by method type, currently/formerly/never in union, number of unions, cohabitation duration (grouped), time since last sex (in days), recent sexual activity, beating justified, ever heard of AIDS, reduce risk of getting HIV, had any STI in last 12 months, ever been tested for HIV, wife justified asking husband to use condom if he has STI, total lifetime number of sex partners, can ask partner to use condom, were selected as predictors of HIV acquisition for females. While, type of place of residence, highest educational level, religion, number of household members, age of household head, times away from home in last 12 months, current contraceptive method, number of injections in last 12 months, respondent circumcised, currently/formerly/never in union, cohabitation duration (grouped), currently working, beating justified, reduce risk of getting HIV, had any STI in last 12 months, wife justified asking husband to use condom if he has STI, total lifetime number of sex partners, were selected as predictors of HIV acquisition for males.

### Algorithm comparison

Model/algorithm’s performance comparison was made based on the LASSO selected features. The comparison was done in two-phases, 1) using the original data and 2) using the SMOTE processed data. Table 2 describes the original and SMOTE data samples for the training, testing and validation sets for the three survey years stratified by sex.

**Table 2:** Description of original data and SMOTE-processed data.

		MALES DATA				FEMALES DATA			
Dataset	Survey Year	Majority class	Minority Class	Total Samples	Ratio	Majority class	Minority Class	Total Samples	Ratio
**Training**	2005–06	7124	1164	8288	6:1	8809	2282	11091	4:1
	2010–11	6684	1186	7870	5:1	8176	2500	10676	3:1
	2015	5739	1194	6933	5:1	7583	2349	9932	3:1
**Training-smote**	2005–06	7124	7124	14248	1:1	8809	8809	17618	1:1
	2010–11	6684	6684	13368	1:1	8176	8176	16352	1:1
	2015	5739	5739	11478	1:1	7583	7583	15166	1:1
**Testing**	2005–06	1782	291	2073	6:1	2202	571	2773	4:1
	2010–11	1672	296	1968	5:1	2045	625	2670	3:1
	2015	1435	299	1734	5:1	1897	587	2484	3:1
**Testing-smote**	2005–06	1782	1782	3564	1:1	2202	2202	4404	1:1
	2010–11	1672	1672	3344	1:1	2045	2045	4090	1:1
	2015	1435	1435	2870	1:1	1897	1897	3794	1:1
**Validation**	2005–06	3312	760	4072	4:1	4345	1604	5949	3:1
	2010–11	3862	733	4595	5:1	5135	1332	6467	4:1
	2015	5044	722	5766	7:1	5876	1521	7397	4:1
**Validation-smote**	2005–06	3312	3312	6624	1:1	4345	4345	8690	1:1
	2010–11	3862	3862	7724	1:1	5135	5135	10270	1:1
	2015	5044	5044	10088	1:1	5876	5876	11752	1:1

Six algorithms were trained and tested using year-specific datasets on the original and SMOTE processed data. Table 3 gives results of the performance of the prediction capabilities of the algorithms. The SMOTE processed data performed better in all metrics than the original data. The precision and the F1 score were extremely low for most of the algorithms in the original data. This means that the original data had many false positives from the low precision and low accuracy based on the low F1 score. Overall, the XGBoost was best performing algorithm in both the original data and SMOTE processed data for all the survey year for each sex.

**Table 3:** Results of classification models in original imbalanced and SMOTE-processed data.

		SMOTE-processed data				Original unbalanced data			
	MALES								
Survey Year	Models	Accuracy %	Precision %	Recall %	F-Score %	Accuracy %	Precision %	Recall %	F-Score %
2005–06	DT	83	87%	78	83	65	25	77	38
2010–11		82	88	75	81	67	29	80	42
2015		81	83	77	80	68	32	74	44
2005–06	EN	64	59	98	71	63	22	65	33
2010–11		64	59	95	72	60	23	69	34
2015		63	59	80	68	57	25	73	37
2005–06	KNN	86	92	78	84	93	79	65	72
2010–11		86	91	80	85	93	81	69	75
2015		82	88	75	81	90	77	63	77
2005–06	RFC	83	79	90	84	70	29	78	42
2010–11		81	78	87	82	66	28	80	42
2015		83	80	86	83	69	32	71	44
2005–06	LIGHTGBM	94	98	90	93	84	45	77	57
2010–11		94	98	89	94	82	45	75	56
2015		92	98	86	91	81	46	71	56
2005–06	XGBOOST	95	95	96	95	93	75	74	74
2010–11		94	95	94	94	92	71	76	73
2015		94	93	94	94	90	72	71	71
	FEMALES								
2005–06	DT	83	86	79	82	72	40	74	52
2010–11		81	84	76	80	70	42	72	53
2015		81	83	79	81	72	45	70	54
2005–06	EN	63	58	88	70	72	39	62	48
2010–11		61	58	80	67	67	38	60	46
2015		59	55	90	69	67	38	63	47
2005–06	KNN	83	90	74	81	89	79	66	72
2010–11		83	89	75	81	89	80	68	74
2015		81	88	72	80	89	81	68	74
2005–06	RFC	84	84	84	84	75	44	70	54
2010–11		81	81	81	81	73	45	68	54
2015		83	83	83	83	75	47	70	56
2005–06	LIGHTGBM	91	96	86	91	82	55	73	63
2010–11		90	94	84	89	79	54	72	62
2015		90	95	85	89	79	54	72	61
2005–06	XGBOOST	93	93	93	93	90	74	76	75
2010–11		92	92	93	92	88	73	77	75
2015		92	92	92	92	88	75	76	76

**Fig 2:**
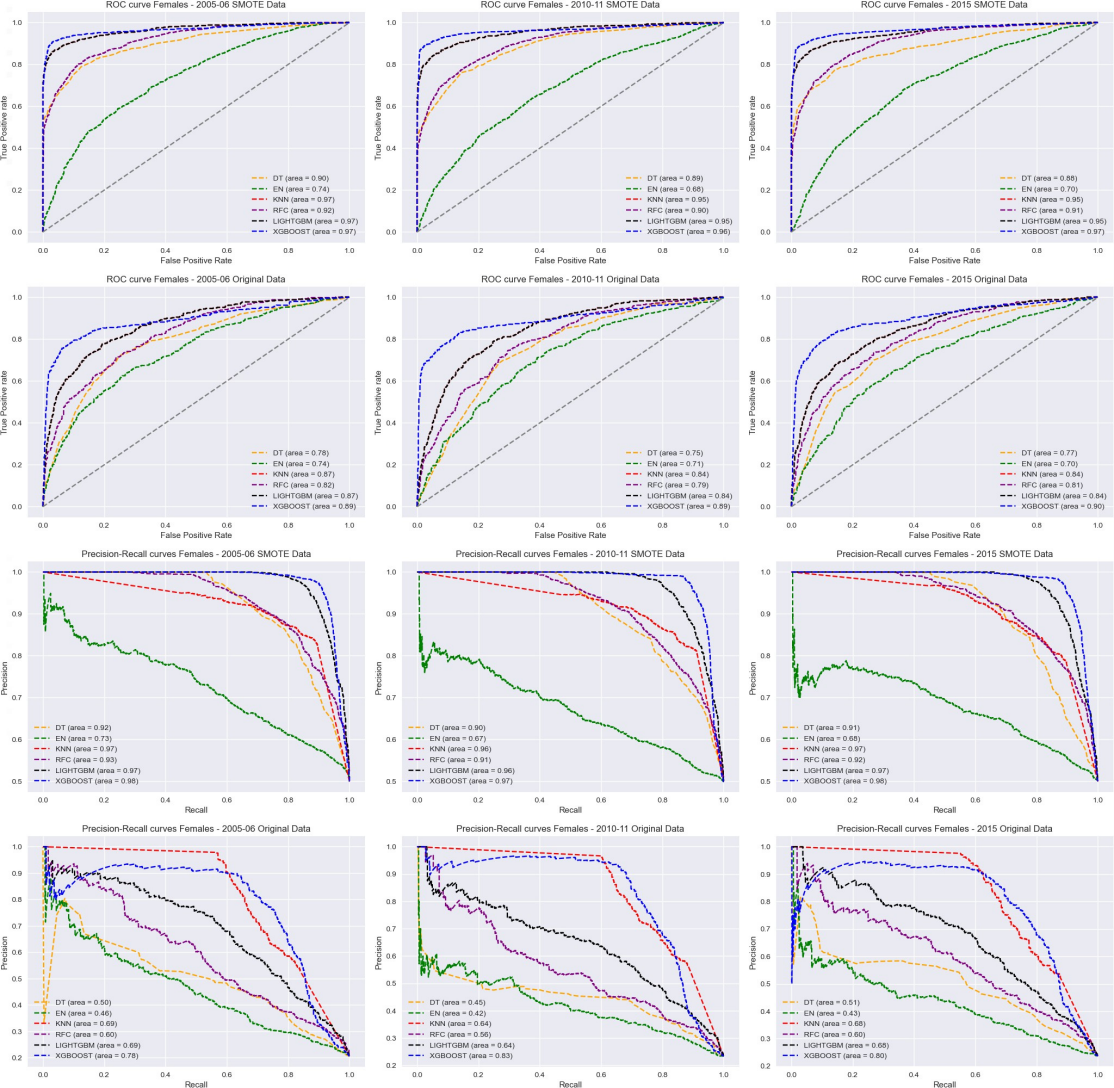
ROC curves (1^st^ and 2^nd^ row) and Precision-Recall curves (3^rd^ and 4^th^row) of the six algorithms for 2005–06 (1^st^ column), 2010–11 (2^nd^ column) and 2015 (3^rd^ column) ZDHS survey for females.

**Fig 3:**
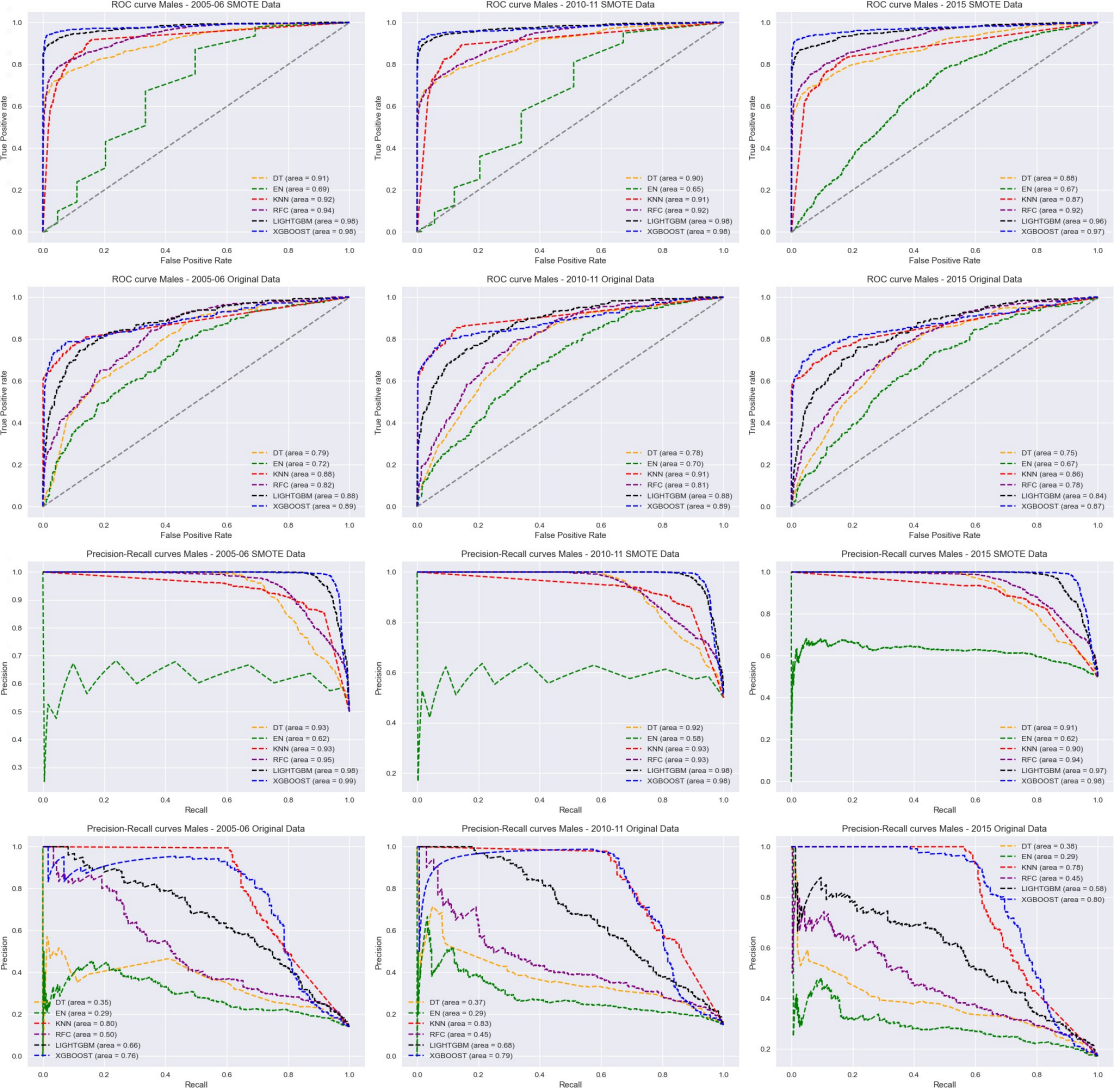
ROC curves (1^st^ and 2^nd^ row) and Precision-Recall curves (3^rd^ and 4^th^row) of the six algorithms for 2005–06 (1^st^ column), 2010–11 (2^nd^ column) and 2015 (3^rd^ column) ZDHS survey for males.

ROC curves and Precision-Recall curves of the six algorithms for all survey years per sex were shown in Figs 2 and 3. The results showed that the XGBoost model had better performance compared to the other 5 models for both the original data and SMOTE processed data. The Elastic Net model performed poorly compared to the other models. Additional ROC and Precision-Recall curves for the six algorithms based on combined survey years can be found in S1 Fig and S2 Fig.

**Fig 4:**
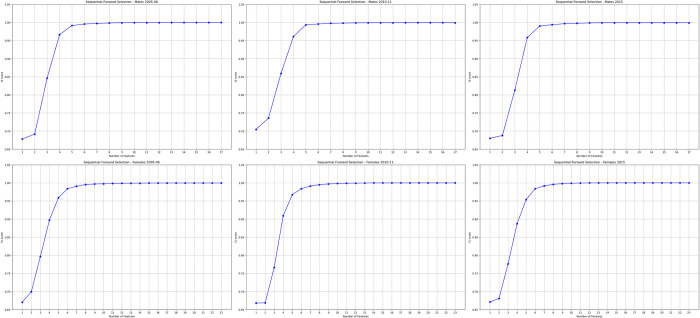
Sequential floating forward selection (SFFS) for males and females.

Subsequent results were obtained utilising the best algorithm model which was the XGBoost. From the features obtained through LASSO, SFFS procedure was implemented on XGBoost to further determine features associated with HIV positivity. Fig 4 shows the saturation limit obtained by the SFFS procedure for selecting variables based on F1 scoring, with 17, 17 and 14 most influential features selected for females, in the 2005–06, 2010–11 and 2015 ZDHS respectively. Whereas, 13, 13, 11 most influential features were selected for males, in the 2005–06, 2010–11 and 2015 ZDHS respectively. The F1 score plateaued at 99.9% for both males and females for all survey years.

**Fig 5:**
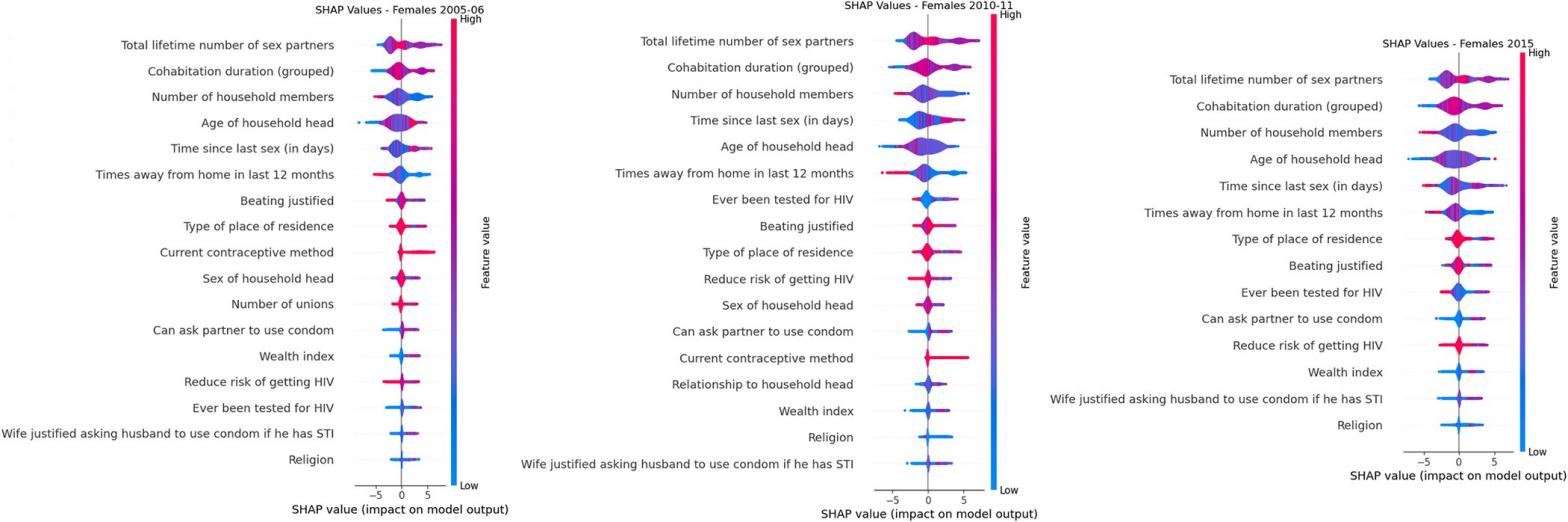
Shapley values for 2005–06, 2010–11 and 2015 Females ZDHS data.

**Fig 6:**
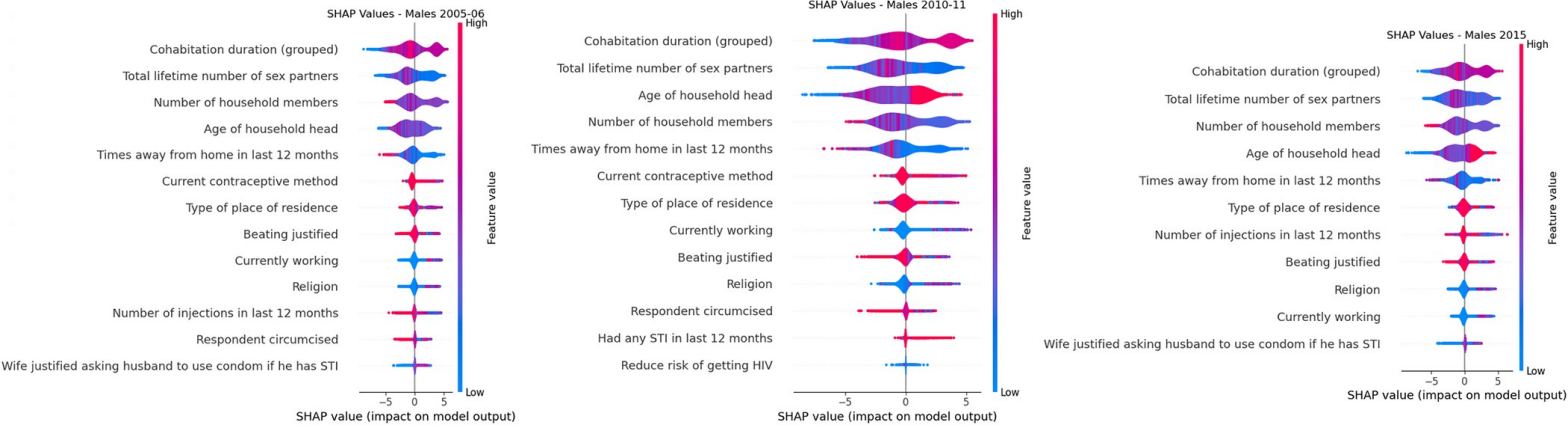
Shapley values for 2005–06, 2010–11 and 2015 Males ZDHS data.

Figs 5 and 6 displays the subset of features most important to predicting an individual’s HIV status following the SFFS procedure. As seen in Figs 5 and 6, the variables are ordered from most important to least important (from highest to lowest Shapley value). According to Fig 5, features for predicting HIV status which appeared throughout for the three survey years for females were: total lifetime number of sex partners, cohabitation duration (grouped), number of household members, age of household head, time since last sex (in days), times away from home in last 12 months, beating justified, can ask partner to use condom, wealth index, reduce risk of getting HIV, wife justified asking husband to use condom if he has STI and religion.

Features for predicting HIV status which appeared throughout for the three survey years for males were: cohabitation duration (grouped), total lifetime number of sex partners, number of household members, age of household head, times away from home in last 12 months, type of place of residence, beating justified, currently working and religion. Identical variables for both sexes throughout the three survey years for predicting HIV status were: total lifetime number of sex partners, cohabitation duration (grouped), number of household members, age of household head, times away from home in last 12 months, beating justified and religion. The two most influential variable for both males and females were total lifetime number of sex partners and cohabitation duration (grouped).

A higher number of total lifetime number of sex partners, more years cohabitating, fewer household members, older age of household head, higher number of days since last sex, fewer times away from home, weather beating was justified or not, living in the rural areas, wearing a condom as current contraceptive method, having a female household head, many unions, can ask partner to use condom, higher wealth index, whether knowing the risk of getting HIV or not, ever been tested, wife justified asking husband to use condom if he has STI and being affiliated to a religion were factors associated with HIV positivity for females in the 2005–06 ZDHS survey. A higher number of total lifetime number of sex partners, more years cohabitating, fewer household members, higher number of days since last sex, younger age of household head, fewer times away from home, never been tested, weather beating was justified or not, living in the rural areas, not knowing the risk of getting HIV, having a female household head, can ask partner to use condom, wearing a condom as current contraceptive method, relationship to household head, higher wealth index, not affiliated to a religion and wife justified asking husband to use condom if he has STI were factors associated with HIV positivity for females in the 2010–11 ZDHS survey. A higher number of total lifetime number of sex partners, more years cohabitating, fewer household members, younger age of household head, fewer number of days since last sex, fewer times away from home, living in the rural areas, beating was justified, never been tested, can ask partner to use condom, not knowing the risk of getting HIV, higher wealth index and wife justified asking husband to use condom if he has STI were factors associated with HIV positivity for females in the 2015 ZDHS survey.

Based on Fig 6, a higher number of years cohabitating, fewer number of total lifetime number of sex partners, fewer household members, younger age of household head, fewer times away from home, not wearing a condom as current contraceptive method, living in the urban areas, beating was justified, currently working, being affiliated to a religion, less number of injections in the last 12 months, respondent not circumcised and wife justified asking husband to use condom if he has STI and were factors associated with HIV positivity for males in the 2005–06 ZDHS survey. A higher number of years cohabitating, fewer number of total lifetime number of sex partners, fewer household members, older age of household head, fewer times away from home, not wearing a condom as current contraceptive method, living in the urban areas, beating was justified, being affiliated to a religion, respondent not circumcised and had an STI in the last 12 months were factors associated with HIV positivity for males in the 2010–11 ZDHS survey. A higher number of years cohabitating, fewer number of total lifetime number of sex partners, fewer household members, older age of household head, fewer times away from home, living in the urban areas, fewer number of injections in the last 12 months, beating not justified, being affiliated to a religion, currently working and wife justified asking husband to use condom if he has STI were factors associated with HIV positivity for males in the 2015 ZDHS survey.

Table 4 shows the odds ratio of HIV infection performed on the selected features obtained through SFFS for females. The results in Table 4 agree with the results shown in Fig 5. For instance, each additional increase in the number of sex partners is associated with a 32% (OR: 1.32, p<0.001), 11% (OR: 1.11, p = 0.04) and 41% (OR: 1.11, p<0.001) increase in the odds of one being HIV infected for 2005–06, 2010–11 and 2015 ZDHS females data, which is corroborated by Fig 5 were total lifetime number of sex partners is associated with HIV positivity as the number of total lifetime sex partners increases for females in all survey years data. Table 4 also shows a protective effect of HIV infection in individuals who live in rural areas than those who reside in urban areas. This, again, is corroborated by Fig 5, were living in urban areas is associated with HIV positivity.

**Table 4:** Odds Ratio of female features selected in XGBoost using SFFS procedure.

Survey	2005–06		2010–11		2015	
Variable	Odds ratio	p-value	Odds ratio	p-value	Odds ratio	p-value
**Total lifetime number of sex partners**	1.315912	<0.001	1.109939	0.037	1.410084	<0.001
**Cohabitation duration (grouped)**						
**Never Married**	ref		ref		ref	
5–9	1.340407	0.011	1.287969	0.034	1.474305	0.002
10–14	1.390041	0.012	1.459357	0.004	1.673866	<0.001
15–19	1.333851	0.032	1.425452	0.018	2.222664	<0.001
20–24	0.8778976	0.046	0.8974349	0.471	1.840237	<0.001
25–29	0.5542526	0.003	0.718883	0.011	1.408705	0.041
30+	0.4943325	0.031	0.5959646	0.048	1.368872	0.031
**Number of household members**	0.9596885	0.02	0.880098	<0.001	0.8523343	<0.001
**Age of household head**	1.105237	0.012	1.126954	<0.001	1.225387	<0.001
**Time since last sex (in days)**	1.002203	<0.001	1.000348	0.287	1.00133	0.001
**Intimate Partner Violence (IPV)**						
no	ref		ref		ref	
yes	1.5837278	0.153	1.614516	0.0466	1.9680338	0.0298
**Type of place of residence**						
urban	ref		ref		ref	
rural	0.8375157	0.251	0.6298531	<0.001	0.6740229	0.013
**Current contraceptive method**						
condom use	ref		ref			
No condom use	2.021359	0.011	5.635275	<0.001		
**Sex of household head**						
male	ref		ref			
female	0.9317121	0.466	0.9885284	0.955		
**Number of unions**						
once	ref					
more than once	2.259846	<0.001				
**Can ask partner to use condom**						
no	ref		ref		ref	
yes	1.042492	0.671	1.294777	0.007	2.029347	<0.001
**Wealth index**						
poor	ref		ref		ref	
poorer	1.315844	0.032	0.8705808	0.285	0.9036212	0.441
middle	1.171341	0.245	0.94288	0.652	1.054575	0.685
richer	1.432668	0.022	0.8276257	0.201	0.7554493	0.092
richest	0.9685689	0.878	0.5113931	<0.001	0.5450553	0.002
**Reduce risk of getting HIV**						
no	ref		ref		ref	
yes	0.917144	0.33	0.8602807	0.018	0.8791833	0.025
**Ever been tested for HIV**						
no	ref		ref		ref	
yes	1.060744	0.509	1.112168	0.273	1.498843	0.045
**Wife justified asking husband to use condom if he has STI**						
no	ref		ref		ref	
yes	0.8672589	0.275	1.159646	0.22	1.029157	0.843
**Religion**						
No religion						
Christian	0.995277	0.974	1.033	0.844	0.7605706	0.101
Apostolic	0.8597532	0.313	0.9342901	0.681	0.7902854	0.156
Other	0.5309193	0.025	0.6997551	0.378	0.5492018	0.162
**Times away from home in last 12 months**			0.999514	0.916	0.9919302	0.06
**Relationship to household head**						
head			ref			
wife			0.7331381	0.166		
daughter			0.7313408	0.283		
daughter-in-law			0.4126077	0.001		
grand-daughter			1.098993	0.864		
sister			1.388966	0.453		
other relative			1.032241	0.904		
not related			1.122641	0.893		

Interestingly, for the 2005–06 and 2010–11 survey data for females, individuals who have been cohabiting for 5–19 years were at higher risk of HIV infection than those who never cohabited. On the other hand, individuals who have more than 19 years of cohabitation have a protective effect of HIV infection than those who have not cohabited. However, this phenomenon changes in the 2015 survey data for females as all individuals who are cohabiting are more at risk of HIV infection than those who are not. HIV positivity was associated with whether one had the knowledge of reducing the risk of HIV transmission or not, according to Fig 5, 2005-6 survey data for females. However, for the 2010–11 and 2015 surveys, HIV positivity was associated with not having any knowledge of reducing the risk of getting HIV. This is similar to the logistic regression in Table 4 results, where having knowledge of reducing the risk of HIV transmission was a protective effect against HIV infection.

Table 5 shows the odds ratio of HIV infection performed on the selected features obtained through SFFS for males. Males who are currently working are less likely to be HIV infected compared to those not working with an odds ratio (OR): 0.75 (p = 0.01) and (OR): 0.71 (p<0.001) for the 2005–06 and 2015 survey years, respectively. If there was intimate partner violence (IPV), the risk of HIV infection was two times more than when there was no intimate partner violence for both males and females, as shown in Tables 4 and 5. As the age of household head increases, the risk of infection within the household increases by 11–20% between 2005–2015 for males and females, as shown in Tables 4 and 5. Males cohabiting are more than 3–9 times at risk of HIV infection than those not cohabiting. This is true for all survey years; as shown in Table 5, the risk increases over the years. The risk of HIV transmission being higher in males is also exhibited in Fig 6, which shows that cohabitation is the most influential feature for males in all survey years associated with HIV positivity.

**Table 5:** Odds Ratio of male features selected in XGBoost using SFFS procedure.

Survey	2005–06		2010–11		2015	
Variable	Odds ratio	p-value	Odds ratio	p-value	Odds ratio	p-value
**Cohabitation duration (grouped)**						
**Never Married**	ref					
0–4	3.347549	<0.001	4.232994	<0.001	4.377312	<0.001
5–9	5.874514	<0.001	7.047588	<0.001	6.530351	<0.001
10–14	7.124908	<0.001	7.865179	<0.001	7.520853	<0.001
15–19	6.792356	<0.001	9.919705	<0.001	8.423026	<0.001
20–24	7.735013	<0.001	9.479581	<0.001	9.519249	<0.001
25–29	3.733829	<0.001	7.960904	<0.001	7.066936	<0.001
30+	6.441015	<0.001	5.548076	<0.001	6.918233	<0.001
**Total lifetime number of sex partners**	1.012441	0.002	1.013705	<0.001	1.014312	<0.001
**Number of household members**	0.9135443	<0.001	0.9221065	<0.001	0.9093683	<0.001
**Age of household head**	1.114831	<0.001	1.117423	<0.001	1.200836	<0.001
**Times away from home in last 12 months**	1.002773	0.425	1.000984	0.747	0.9966006	0.234
**Current contraceptive method**						
condom use	ref		ref			
no condom use	1.237349	0.155	2.370684	<0.001		
**Type of place of residence**						
urban	ref		ref		ref	
rural	0.9331089	0.484	0.9045717	0.324	0.871984	0.119
**Intimate Partner Violence (IPV)**						
no	ref		ref		ref	
yes	1.613896	0.288	1.882546	0.0236	1.780742	0.01
**Currently working**						
no	ref		ref		ref	
yes	0.7523402	0.006	0.959925	0.678	0.7097387	<0.001
**Religion**						
No religion	ref		ref		ref	
Christian	0.7597485	0.01	0.8094892	0.055	0.7136371	0.002
Apostolic	0.710367	0.008	0.8097574	0.081	0.8003416	0.048
Other	0.9252984	0.592	0.9331523	0.703	1.322868	0.126
**Number of injections in last 12 months**	1.170525	<0.001			1.007539	0.252
**Respondent circumcised**						
no	ref		ref			
yes	1.156246	0.258	0.9399439	0.663		
**Wife justified asking husband to use condom if he has STI**						
no	ref				ref	
yes	1.564861	0.003			1.424217	0.017
**Had any STI in last 12 months**						
no			ref			
yes			2.305245	<0.001		
**Reduce risk of getting HIV**						
no			ref			
yes			0.8435852	0.167		

## Discussion

Although there have been a few studies utilising machine learning techniques to predict HIV in the generalised HIV pandemic, this is, to our knowledge, the first one in Zimbabwe using routinely collected survey data. This study’s primary goal was to determine the most prevalent risk factors for HIV infection and the predicted accuracy of machine learning models based on these risk factors.

By using socio-demographic factors obtained from three ZDHS, this study was able to predict the HIV status of individuals. After comparison of different algorithms, XGBoost was the best algorithm to predict HIV status. The algorithm was able to determine the most predictive features/variables of HIV infection common to both sexes. Using SHAP plots, this study additionally evaluated the direction of the relationship between HIV infection and the predictive variables.

The total number of sexual lifetime partners was the main influential feature for females, and cohabitation duration for males in all survey years. Previous studies have shown that the total number of sexual partners is a risk factor for HIV infection [28–31]. This was in agreement with our results which indicated that the higher the total number of sexual partners an individual has, the more at risk of HIV infection. To add to the same evidence, a study by Armstrong et al. [32] indicated that sexual partner number was an important HIV risk measure. Our findings also showed that cohabitation was a risk factor of HIV positivity. This finding can be corroborated by other studies which reported that most heterosexual rates of HIV transmission take place within cohabiting or married couples [33–37].

Numerous studies have shown that child-head families are more at risk of HIV infection [38–41]. This is contrary to our findings, as our results indicated that households headed by older individuals are more at risk of HIV infection. This may be attributed to the fact that the older the household head is, the less financially stable they are, hence their only source of income for that family might be from their pension fund, which might not be adequate, resulting in the other household members seeking financial assistance from risky behaviours [42,43].

In addition, living in urban areas was found to increase the probability of HIV positivity. This was corroborated by other studies [44–46], which reported that people in urban settings had a greater chance of contracting HIV than people in rural areas.

Our results indicated a higher risk of HIV infection if there was pro-intimate partner violence. To support this evidence, research done among ever-married and cohabiting women in Zimbabwe by Henderson et.al [47] studied the relationship between intimate partner violence and HIV status, and they found out that women who had been victims of any kind of intimate partner violence were more likely to be HIV positive. The study also commented that the patriarchal and hypermasculist culture in Zimbabwe contributed to the likelihood of HIV infection. Further research utilising the ZDHS 2005–06 indicated that approximately six out of every ten women who reported experiencing some form of violence in their lifetimes had a much higher risk of being HIV-positive than women who had not suffered any physical or sexual abuse [48].

To ensure that a substantial percentage of persons tested are HIV positive, a high yield is necessary when targeted HIV case-finding procedures are used to boost testing’s cost-effectiveness. The potential for further behavioural-based case-finding techniques to improve or supplement current focused case-finding techniques like index testing is still unknown. For some limited resource conditions and the planned test coverage, it may be necessary to modify the acceptable cut-offs for sensitivity and PPV thresholds.

By better identifying those at high risk of contracting HIV, machine learning algorithms have the potential to enhance the implementation of pre-exposure prophylaxis (PrEP). As part of an inclusive approach to PrEP, which has gone from development to implementation, programs can utilise these algorithms to spark discussions about PrEP. However, the limited number of variables accessible to us for this study constituted one of its drawbacks. We were unable to determine the impact of variables, including viral load, health care spending, HIV-risk groups, and other HIV-related interventions. The data also contained missing values, which necessitated making assumptions about their unpredictable nature and applying intrinsically flawed imputation techniques. Finally, several variables were self-reported and, as a result, were vulnerable to recall bias and social desirability.

Our technique for predictor identification primarily adapts from Orel’s [49] and Mutai’s [2] methodology, which also chose the XGBoost algorithm as the best. Contrarily, our findings demonstrate distinct predictors from those discovered in Orel’s and Mutai’s study. Considering that our study was more specific to Zimbabwe, this might have been the reason why results from Mutai’s study were not similar to our findings. Additionally, Orel’s population was based on Eastern Africa, which might have different epidemiological factors from the sub-Saharan Africa, therefore making the results statistically incomparable.

## Conclusion

Our findings may help with social-behavioural HIV detection and improve screening procedures in limited resource settings. In addition, the amount of information needed to identify key populations in Zimbabwe will be significantly reduced by features/variables which have been identified in this study through machine learning. Adaptation of HIV screening methods that more effectively target the adult population, those with multiple partners, those who are frequently away from home, those who reside in urban areas, those who are not currently working and other risk factors associated with HIV positivity are needed. Programmes targeted at HIV testing could incorporate machine learning approaches to adequately and effectively identify high-risk individuals. However, to improve the machine learning approach, further research is required to integrate and implement them in a real-world primary care context. In addition to other risk reduction techniques, machine learning may aid in identifying those who might require PrEP.

## Supporting information

S1 Table Male and Female predictors variables considered in variable selection. (DOCX)

S1 Fig ROC Curves for SMOTE and ORIGINAL data of males and females combined survey years data. (TIF)

S2 Fig Precision-Recall Curves for SMOTE and ORIGINAL data of males and females combined survey years data. (TIF)
