# Supplementary material for: Predicting HIV infection in the decade (2005–2015) pre-COVID-19 in Zimbabwe: A supervised classification-based machine learning approach
Source: PLOS Digit Health. 2023 Jun 7;2(6):e0000260. doi: 10.1371/journal.pdig.0000260 (PMC10246851; doi:10.1371/journal.pdig.0000260)
Supplement: S1 Table — (DOCX) [file pdig.0000260.s001.docx]

**S1 Table: Male and Female predictors variables.**

|  | Male Features | Female Features |
| --- | --- | --- |
| 1.  2.  3.  4.  5.  6.  7.  8.  9.  10.  11.  12.  13.  14.  15.  16.  17.  18.  19.  20.  21.  22.  23.  24.  25.  26.  27.  28.  29.  30.  31.  32. | Current age  Type of place of residence  Highest educational level  Religion  Number of household members  Relationship to household head  Sex of household head  Age of household head  Times away from home in last 12 months  Wealth index  Current contraceptive method  Current contraceptive by method type  Number of injections in last 12 months  Respondent circumcised  Currently/formerly/never in union  Number of unions  Age at first cohabitation  Cohabitation duration (grouped)  Time since last sex (in days)  Age at first sex (imputed)  Recent sexual activity  Wife justified refusing sex: husband has other women  Currently working  Type of earnings  Beating justified  Reduce risk of getting HIV  Had any STI in last 12 months  Number of sex partners, including spouse, in last 12 months  Ever been tested for HIV  Paid for sex in last 12 months  Wife justified asking husband to use condom if he has STI  Total lifetime number of sex partners | Current age  Type of place of residence  Highest educational level  Religion  Number of household members  Relationship to household head  Sex of household head  Age of household head  Times away from home in last 12 months  Wealth index  Ever had a terminated pregnancy  Current contraceptive method  Current contraceptive by method type  Number of injections in last 12 months  Currently/formerly/never in union  Number of unions  Age at first cohabitation  Cohabitation duration (grouped)  Time since last sex (in days)  Age at first sex (imputed)  Recent sexual activity  Currently working  Beating justified  Ever heard of AIDS  Reduce risk of getting HIV  Had any STI in last 12 months  Number of sex partners, including spouse, in last 12 months  Ever been tested for HIV  Wife justified asking husband to use condom if he has STI  Total lifetime number of sex partners  Can respondent refuse sex  Can ask partner to use condom |
