# Supplementary figures and images for: Predicting HIV infection in the decade (2005–2015) pre-COVID-19 in Zimbabwe: A supervised classification-based machine learning approach
Source: PLOS Digit Health. 2023 Jun 7;2(6):e0000260. doi: 10.1371/journal.pdig.0000260 (PMC10246851; doi:10.1371/journal.pdig.0000260)

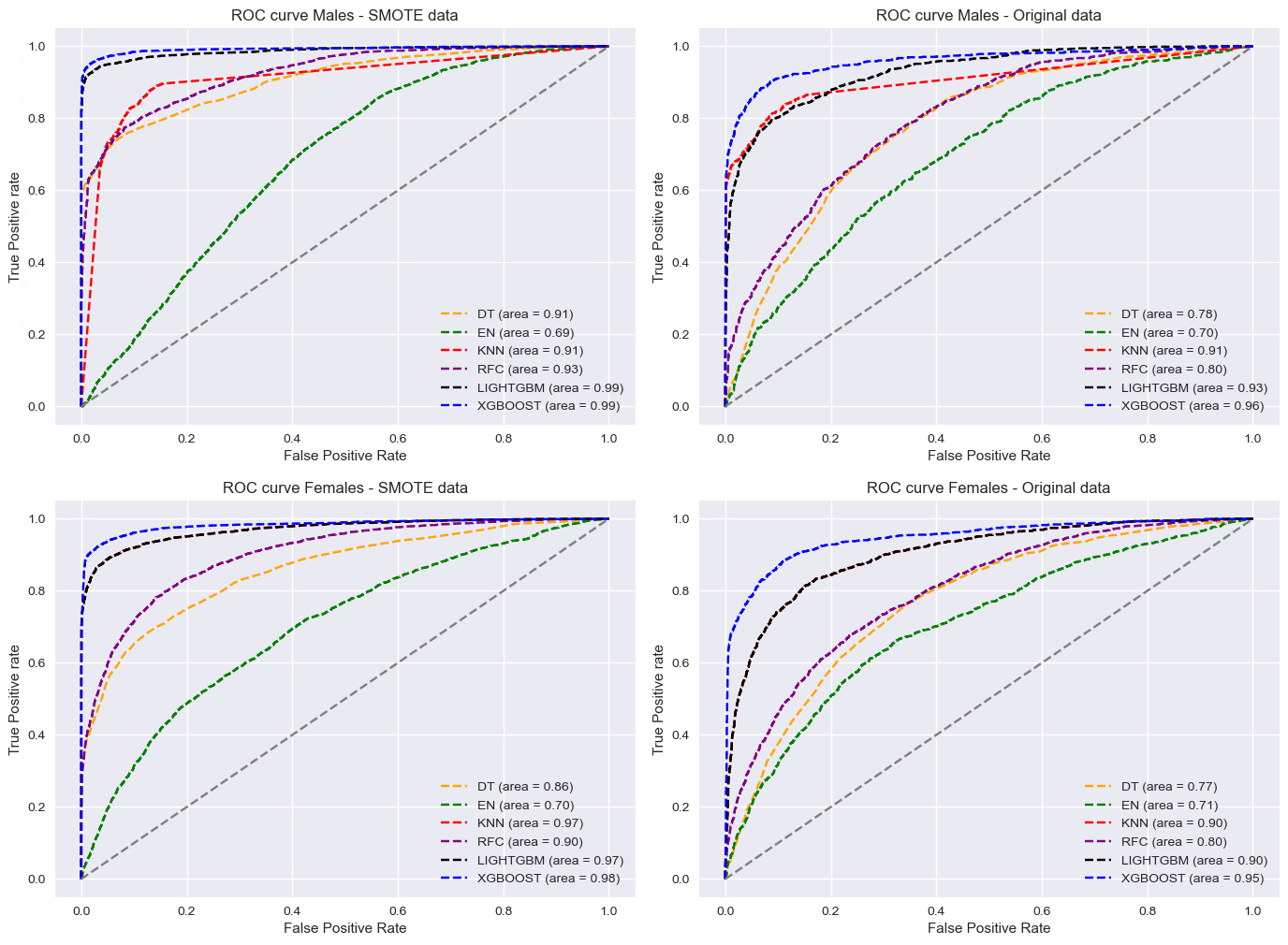

Supplement: S1 Fig — (TIF) [file pdig.0000260.s002.tif]

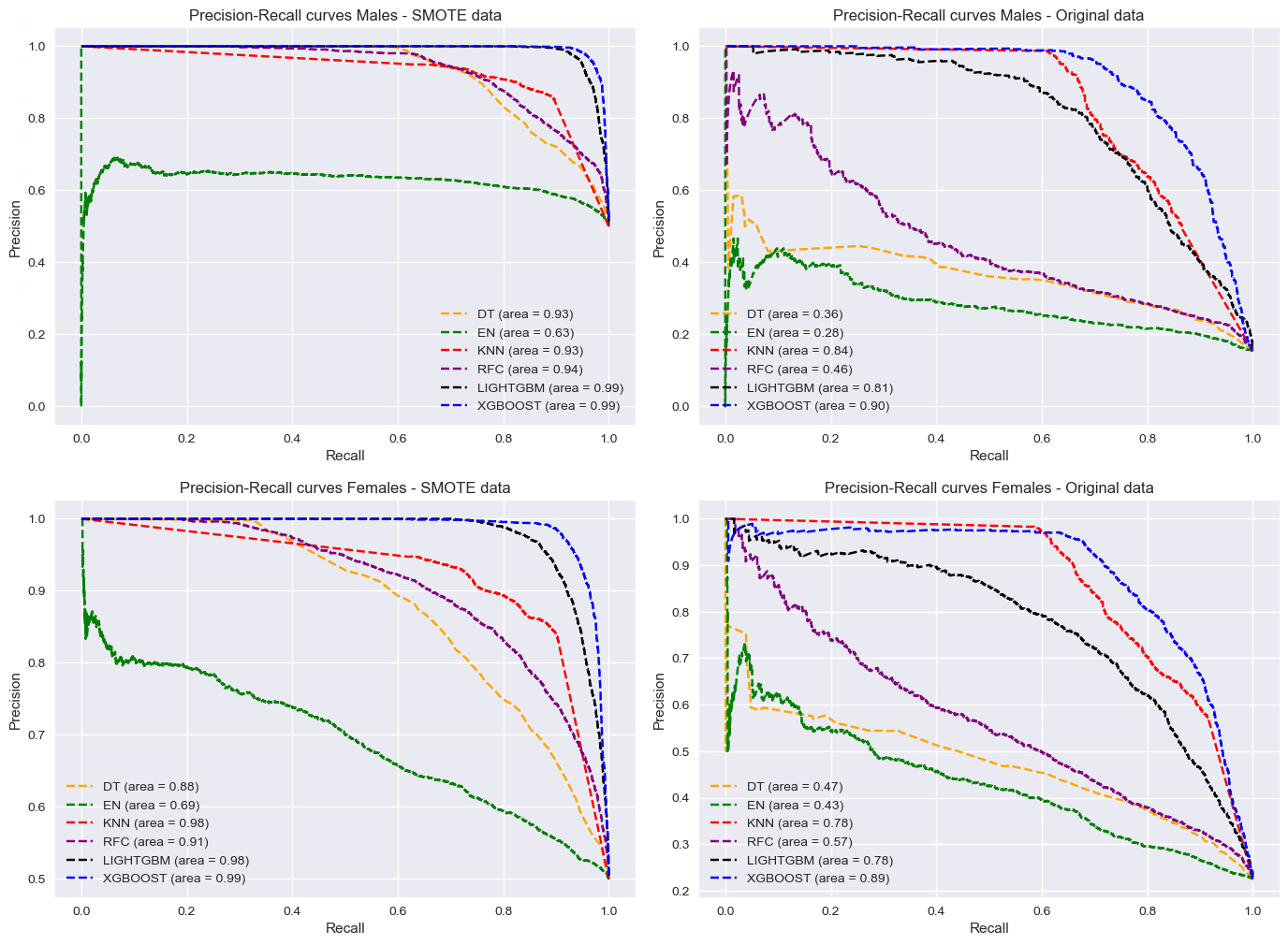

Supplement: S2 Fig — (TIF) [file pdig.0000260.s003.tif]
